# Role of Closed Suction Drains in Obese Patients Undergoing Major Urological Procedures: A Prospective Comparative Study

**DOI:** 10.7759/cureus.91955

**Published:** 2025-09-10

**Authors:** Firdous Ahmad Beigh, Mudasir Ahmad Tantray, Sajad A Malik, Tufeel Ahmad Khan, Sajjad A Para, Abdul Khawaja, Saqib Mehdi, Arif Hamid, Saundarya Kumar Verma, Syed Shakeeb Arsalan

**Affiliations:** 1 Urologic Oncology, Sher-i-Kashmir Institute of Medical Sciences, Srinagar, IND; 2 Urology, Sher-i-Kashmir Institute of Medical Sciences, Srinagar, IND

**Keywords:** closed suction drains, obesity, postoperative morbidity, radical cystectomy, radical nephrectomy, radical prostatectomy, seroma formation, subcutaneous fat thickness, wound complications

## Abstract

Background: Obese patients undergoing major urological procedures, such as open radical prostatectomy, open radical cystectomy, and open radical nephrectomy, are at heightened risk of postoperative wound complications, particularly seroma formation, due to increased subcutaneous fat thickness. Closed suction drains in the subcutaneous tissue are hypothesized to mitigate these complications by reducing dead space and fluid accumulation. This prospective, randomized, and comparative study investigates the efficacy of subcutaneous closed suction drains in reducing wound complications in obese patients undergoing these procedures.

Methods: Sixty obese patients (body mass index (BMI) >32 kg/m^2^, subcutaneous fat thickness ≥3 cm) scheduled for elective open radical prostatectomy, open radical cystectomy, or open radical nephrectomy were randomized into two groups: study (n = 30, with subcutaneous closed suction drains) and control (n = 30, without drains). Exclusion criteria included intraoperative spillage, sepsis, diabetes, malignancy beyond the primary urological condition, steroid therapy, or other factors affecting wound healing. Surgical techniques and wound closure were standardized, with prophylactic antibiotics administered. Subcutaneous fat thickness was measured intraoperatively. Outcomes, including seroma formation, wound infection, hematoma, and wound dehiscence, were assessed clinically over a one-month follow-up. Statistical analysis used chi-square tests, Fisher’s exact tests, and logistic regression to evaluate associations.

Results: Seroma formation occurred in four (13.33%) patients in the study group and 14 (46.67%) in the control group (p = 0.004). The odds ratio (OR) for seroma formation without drains was 5.6 (95% CI: 1.6-19.8) for subcutaneous fat thickness of 3-3.9 cm and 8.9 (95% CI: 2.1-37.4) for 4-5 cm. Most seromas (75% in the study group, 78.57% in the control group) presented within the first postoperative week. Wound infection occurred in one (3.33%) control group patient (p = 0.99). No hematomas or wound dehiscence were reported. Logistic regression confirmed subcutaneous fat thickness as a significant predictor of seroma formation (p = 0.01).

Conclusion: Prophylactic closed suction drains significantly reduce seroma formation and surgical site infection (SSI) in obese patients in obese patients undergoing major urological procedures, potentially decreasing postoperative morbidity and healthcare costs. Routine use is recommended in this population, with further research needed to optimize drain duration and evaluate efficacy in non-obese patients or minimally invasive procedures.

## Introduction

Obesity, defined as a body mass index (BMI) >30 kg/m^2^, is a well-established risk factor for postoperative wound complications, particularly in procedures involving large incisions, such as open radical prostatectomy, open radical cystectomy, and open radical nephrectomy. These major urological surgeries often result in significant disruption of subcutaneous tissue, leading to dead space, lymphatic disruption, and fluid accumulation, which predispose to seromas, hematomas, and infections [1]. Seromas, in particular, increase morbidity by prolonging recovery, necessitating additional interventions, and increasing healthcare costs.

Closed suction drains in the subcutaneous tissue are designed to evacuate serous fluid, blood, and debris, thereby reducing dead space and promoting wound healing. While their use is common in certain surgical fields, such as general surgery and gynecology [1-3], evidence in urological procedures remains sparse. Studies in other disciplines, such as cholecystectomy [2] and cesarean delivery [4], suggest that drains reduce seroma formation in obese patients [5,6], but conflicting reports exist regarding their impact on infection rates [7,8]. The anatomical considerations in urological surgeries, such as the pelvic and retroperitoneal spaces, may influence the efficacy of drains due to varying degrees of tissue trauma and lymphatic disruption.

This study aims to evaluate the role of prophylactic closed suction drains in reducing wound complications, primarily seroma formation, in obese patients undergoing major urological procedures. By focusing on open radical prostatectomy, cystectomy, and nephrectomy, we address a gap in the literature and provide evidence to guide clinical practice in urological surgery.

## Materials and methods

Study design and population

This prospective, randomized, comparative study was conducted at the Department of Urology, Sher-i-Kashmir Institute of Medical Sciences (SKIMS), Srinagar, from January 2023 to June 2024. Sixty obese patients (BMI >32 kg/m^2^, with Grade II obesity per WHO classification; subcutaneous fat thickness 3-5 cm), scheduled for elective open radical prostatectomy, open radical cystectomy, or open radical nephrectomy, were enrolled. Patients were randomized into two groups (study, n = 30; control, n = 30) using systematic random sampling, with alternate allocation to study (drain) and control (no drain) groups by the lead consultant.

Exclusion criteria included intraoperative spillage of bladder, prostate, or renal contents; evidence of sepsis; diabetes mellitus; advanced malignancy beyond the primary urological condition; steroid therapy; radiotherapy; hepatic or renal insufficiency; or any condition impairing wound healing. Patients aged 20-60 years of either sex were eligible.

The Instutional Ethical Committee of SKIMS, Srinagar, issued approval SIMS 135/IEC-SKIMS/2023-92.

Surgical procedure

Surgeries were performed by experienced urologists using standardized techniques. Open radical prostatectomy and cystectomy used a lower midline incision, while nephrectomy used a flank incision. Electrocautery was employed for hemostasis. Subcutaneous fat thickness was measured intraoperatively using a sterile ruler at the deepest point of the incision. Wound closure involved continuous absorbable sutures (1-0 PDS) for the fascial layer and staples for the skin, uniform across groups. In the study group, a 14F closed suction drain was placed in the subcutaneous tissue, exiting via a separate stab incision and connected to a vacuum drainage system. Drains were removed after 48 hours, based on prior studies [1]. Prophylactic antibiotics (cefaperazone-sulbactam 1.125 g IV, 30 minutes preoperatively and 12 hourly postoperatively for three to five days) were administered to all patients.

Outcome assessment

Primary outcomes included seroma formation, defined as serous discharge or a soft, fluctuant bulge confirmed by aspiration of serous fluid. Secondary outcomes were wound infection (erythema and cellulitis), hematoma, and wound dehiscence, thereby increasing hospital stay and outpatient visits. Patients were evaluated daily during hospitalization (discharged on postoperative days 3-7) and weekly in the outpatient department for 4 weeks. Seromas were managed with aspiration through the wound and antiseptic dressings.

Statistical analysis

Data were analyzed using IBM SPSS Statistics for Windows, Version 25.0 (released 2017, IBM Corp., Armonk, NY). Categorical variables (e.g., seroma incidence) were compared using chi-square or Fisher’s exact tests. Logistic regression assessed the association between subcutaneous fat thickness and seroma formation, adjusting for age, sex, and procedure type. A p-value <0.05 was considered significant. The sample size (n = 60) was calculated based on an expected seroma rate of 30% in the control group and 10% in the study group (80% power, α = 0.05), derived from prior studies [1].

## Results

Patient characteristics

The study and control groups were comparable for age (mean 48.5 ± 8.2 vs. 47.8 ± 7.9 years, p = 0.72), sex (male: female ratio 4:1 in both groups), and BMI (range 32-34). Procedure distribution was balanced: 10 prostatectomies, 12 cystectomies, and eight nephrectomies per group. Subcutaneous fat thickness ranged from 3 to 5 cm, with equitable distribution.

Seroma formation

Seroma was the primary complication. Overall, four (13.33%) study group patients and 14 (46.67%) control group patients developed seromas (p = 0.004, OR = 5.6, 95% CI: 1.6-19.8), as shown in Table 1.

**Table 1:** Overall distribution with respect to seroma development Fisher's exact test was used for the calculation of of the p-value.

Group	Seroma	No seroma	Total	p-value
					Study	4 (13.33%)	26 (86.67%)	30 (100%)	0.04
Control	14 (46.67%)	16 (53.33%)	30 (100%)						

Seroma rates between the study and control groups stratified by fat thickness, as presented in Table 2.

**Table 2:** Seroma development with respect to subcutaneous fat thickness Fisher's exact rest was used for the statistical analysis and calculation of the p-value.

Subcutaneous fat thickness	Study		Control		p-value
	Seroma	No seroma	Seroma	No seroma	
3-3.9 cm	1 (6.67%)	14 (93.33%)	6 (40%)	9 (60%)	0.04
4-5 cm	3 (20%)	12 (80%)	8 (53.33%)	7 (46.67%)	0.03
Total	15		15		

Logistic regression confirmed subcutaneous fat thickness as a significant predictor of seroma formation (p = 0.01), with no significant effect of age (p = 0.62), sex (p = 0.78), or procedure type (p = 0.45).

Timing of seroma presentation

The timing of seroma formation between the study and control groups on a weekly basis is presented in Table 3. No seromas were reported in the fourth week.

**Table 3:** Seroma development with respect to duration after surgery Fisher's exact test was used for the statistical analysis and calculation of the p-value.

Group	First week	Second week	Third week	Fourth week	Total
						Study	3 (75%)	1 (25%)	0 (0%)	0 (0%)	4 (100%)
Control	11 (78.57%)	2 (14.29%)	1 (7.14%)	0 (0%)	14 (100%)						

Six patients in the control group (20%) developed a wound infection (p-value = 0.02, Fisher’s exact test). No hematomas or wound dehiscence were observed in either group. All seromas resolved with aspiration and dressings, with no rehospitalizations. Patients with surgical site infections (SSIs) in the control group were managed with local wound dressings (Table 4).

**Table 4:** Comparison of surgical site infections (SSIs) Fisher’s exact test was used for the statistical analysis.

Group	Number of patients (%)	Patients with SSIs (%)	p-value
Study limb	30 (100%)	0 (0%)	0.02
Control limb	30 (100%)	6 (20%)	

**Figure 1:**
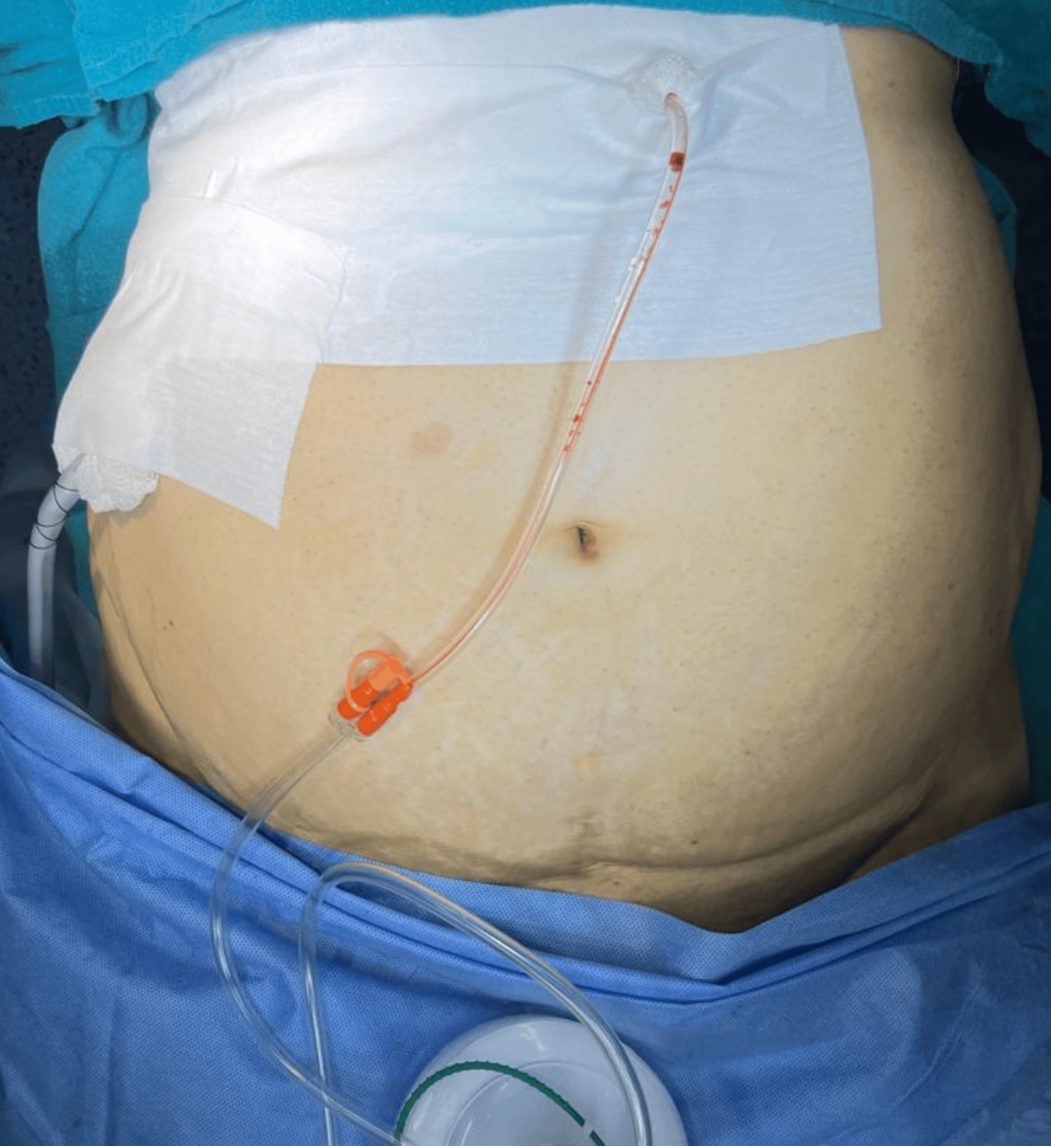
POD 0 of right radical nephrectomy with IVC thrombectomy. Suction drain placed on the right along with the ADK drain. POD: postoperative day, IVC: inferior vena cava, ADK: abdominal drainge kit, ASD: antiseptic dressing

**Figure 2:**
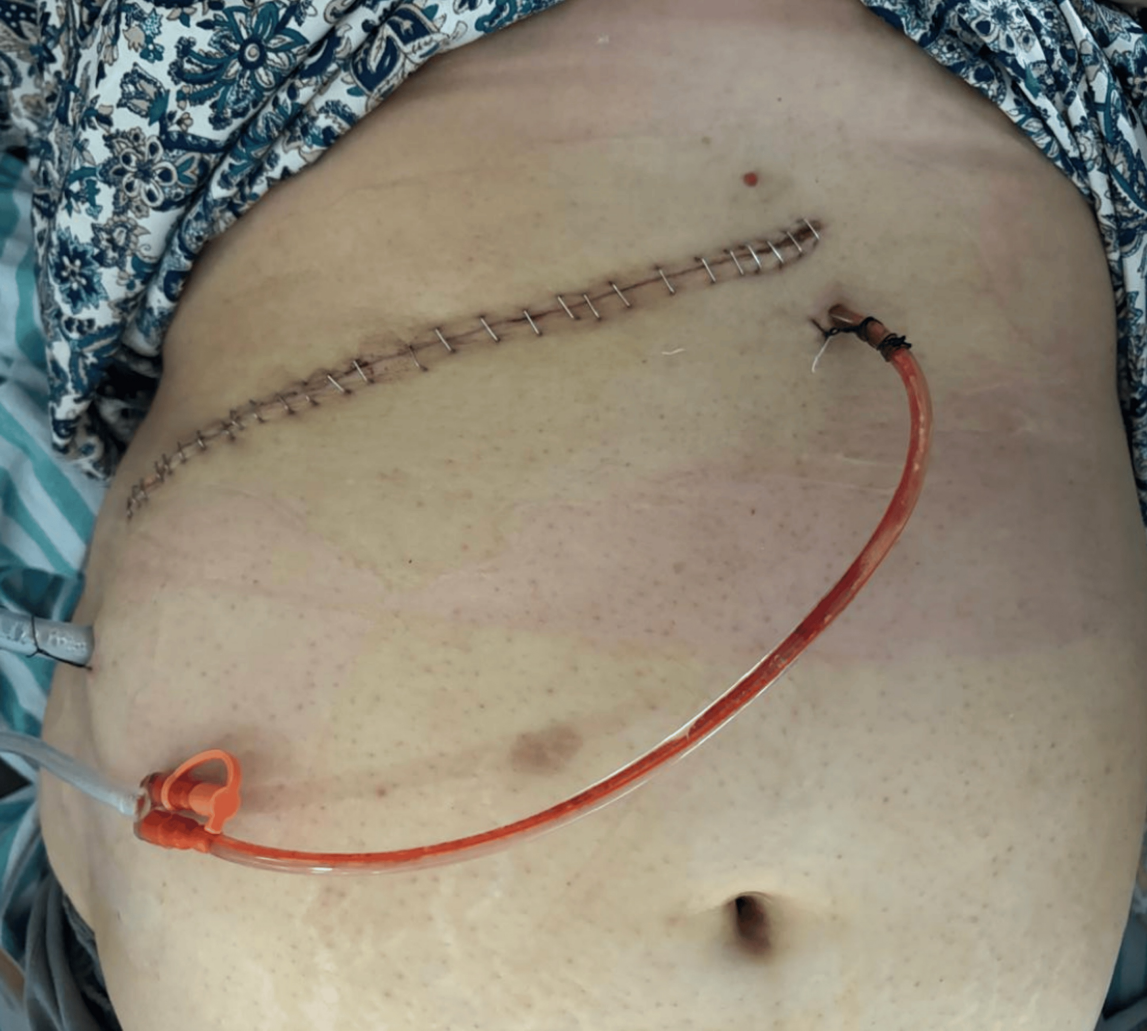
Right radical nephrectomy with IVC thrombectomy wound on POD 4. No signs of inflammation. IVC: inferior vena cava, POD: postoperative day

Figure 1 reveals a post-radical nephrectomy with IVC thrombectomy wound with an antiseptic dressing (ASD) in place. A suction drain was placed in addition to an abdominal drainage kit (ADK). A wound was examined on POD 4 with no signs of inflammation, and the wound edges were nicely epithelialized (Figure 2). The suction drain was removed on POD 5. 

## Discussion

This study demonstrates that prophylactic closed suction drains significantly reduce seroma formation in obese patients undergoing major urological procedures, with a seroma rate of 13.33% in the drain group versus 46.67% in controls. These findings align with Chowdri et al.’s study on cholecystectomy [1], which reported an 8.8% versus 33.6% seroma rate, and Gallup et al.’s gynecological study [3], which noted reduced complications with drains. The higher seroma risk with increased subcutaneous fat thickness (OR = 5.6 for 3-3.9 cm, 8.9 for 4-5 cm) corroborates Soper et al.’s findings [9], emphasizing the role of adipose depth in wound complications.

The absence of increased infection rates with drains contradicts older studies [8], suggesting bacterial migration. Modern surgical techniques, improved sterilization, and prophylactic antibiotics likely mitigate this risk. The low infection rate (20% in the controls, 0% in the study group) in the study group supports its use in such patients, consistent with Yeleti SA et al. [10] of drain use. The lack of hematomas or dehiscence may reflect careful patient selection and standardized closure techniques, unlike Worcester’s study [4], which reported higher rates of these complications in cesarean patients without drains.

Clinical implications

The significant reduction in seroma formation suggests that closed suction drains should be considered standard practice in obese patients undergoing open urological procedures. Seromas increase outpatient visits, interventions, and patient distress, contributing to healthcare costs. By reducing seroma incidence and SSI, drains may improve patient satisfaction and recovery time. However, the 48-hour drain duration, based on prior protocols [1], may not be optimal. Extended drainage could further reduce late-presenting seromas, as seen in 25% of study group cases occurring in the second week.

Comparison with urological literature

Urological procedures differ from general surgical or gynecological operations due to deeper tissue planes and lymphatic disruption, particularly in cystectomy and nephrectomy. Albino et al. [11] noted that obesity increases wound complications in body contouring after massive weight loss, a scenario analogous to urological incisions in obese patients. Our findings extend this to urology, suggesting drains are particularly beneficial in patients with subcutaneous fat thickness of 3-5 cm, where dead space and lymphatic disruption are pronounced.

Limitations and future directions

The sample size (n = 60) limits statistical power, although significant results were achieved. The focus on obese patients (BMI 32-34) excludes non-obese and morbidly obese populations, limiting generalizability. The study did not assess minimally invasive urological procedures (e.g., laparoscopic or robotic-assisted), where smaller incisions may alter drain efficacy. Future research should explore optimal drain duration, cost-effectiveness, and applicability to laparoscopic procedures. Patient-reported outcomes, such as pain or satisfaction, could further inform clinical guidelines.

Economic and psychological impact

Seroma and SSI management increased hospital stay and outpatient visits in the control group, likely elevating costs and patient anxiety. Drains may reduce these burdens, supporting their routine use in high-risk patients like obese ones [12,13,14].

## Conclusions

Prophylactic closed suction drains significantly reduce seroma formation and SSIs in obese patients undergoing open radical prostatectomy, cystectomy, or nephrectomy, with 5.6-fold lower odds in patients with drains. Routine use is recommended in this population to decrease morbidity, outpatient interventions, and healthcare costs. Further studies should optimize drain duration, evaluate non-obese patients, and assess minimally invasive approaches.
